# Diagnostic utility of the soluble triggering receptor expressed on myeloid cells-1 in bronchoalveolar lavage fluid from patients with bilateral lung infiltrates

**DOI:** 10.1186/cc6770

**Published:** 2008-01-19

**Authors:** Jin Won Huh, Chae-Man Lim, Younsuck Koh, Yeon Mok Oh, Tae Sun Shim, Sang Do Lee, Woo Sung Kim, Dong Soon Kim, Won Dong Kim, Sang-Bum Hong

**Affiliations:** 1 Department of Pulmonary and Critical Care Medicine, Ilsan Paik Hospital, Inje University, 2240, Daehwa-dong, Ilsanseo-gu, Goyang-si, Gyeonggi-do, 411-706, Korea; 2 Division of Pulmonary and Critical Care Medicine, Asan Medical Center, College of Medicine, University of Ulsan, 388-1, Pungnap-dong, Songpa-gu, Seoul, Korea

## Abstract

**Background:**

Differential diagnosis of patients with bilateral lung infiltrates remains a difficult problem for intensive care clinicians. Here we evaluate the diagnostic role of soluble triggering receptor expressed on myeloid cells-1 (sTREM-1) in bronchoalveolar lavage (BAL) specimens from patients with bilateral lung infiltrates.

**Methods:**

We conducted a prospective observational study on 80 patients with bilateral lung infiltrates with clinical suspicion of infectious pneumonia. Patients were categorized into three groups: bacterial or fungal infection, intracellular or viral infection, and noninfectious inflammatory disease. sTREM-1 concentrations were measured, and BAL fluid and Clinical Pulmonary Infection Score (CPIS) were analyzed.

**Results:**

The sTREM-1 concentration was significantly increased in patients with bacterial or fungal pneumonia (*n *= 29, 521.2 ± 94.7 pg/ml), compared with that in patients with viral pneumonia, atypical pneumonia or tuberculosis (*n *= 14, 92.9 ± 20.0 pg/ml) or noninfectious inflammatory disease (*n *= 37, 92.8 ± 10.7 pg/ml). The concentration of sTREM-1 in BAL fluid, but not CPIS, was an independent predictor of bacterial or fungal pneumonia, and a cutoff value of more than 184 pg/ml yielded a diagnostic sensitivity of 86% and a specificity of 90%.

**Conclusion:**

The sTREM-1 level in BAL fluid from patients with bilateral lung infiltrates is a potential marker for the differential diagnosis of pneumonia due to extracellular bacteria.

## Introduction

Differential diagnosis of patients with bilateral lung infiltrates remains a difficult problem for intensive care clinicians. Diverse presumptive clinical diagnoses of bilateral lung infiltrates include severe pneumonia induced by bacteria, virus, fungi or tuberculosis, and noninfectious inflammatory diseases caused by collagen vascular disease associated with interstitial lung disease, acute exacerbation of interstitial lung disease, pulmonary edema, acute respiratory distress syndrome or drug-induced lung disease [1]. Notably, several noninfectious processes other than pneumonia lead to fever, leukocytosis, hypoxemia, purulent tracheal secretions, and diffuse pulmonary infiltrates. To enhance the specificity of clinical criteria for diagnosing ventilator-associated pneumonia, the Clinical Pulmonary Infection Score (CPIS) was introduced, which showed a high diagnostic accuracy for ventilator-associated pneumonia in some cases [2,3]. Gibot and colleagues also showed that CPIS could differentiate between patients with and without pneumonia [4]. However, the utility of CPIS remains to be validated, particularly in patients with bilateral infiltration [5]. The need for serology and microbiological tests could delay differential diagnosis for 48 to 72 hours, and the positive culture rate may be low [6-8].

Triggering receptors expressed on myeloid cells (TREMs) are members of the immunoglobulin (Ig) superfamily, a critical component of the innate immune defense system against infection [9,10]. TREM-1 expression is upregulated by extracellular bacteria and fungi but is weak in mycobacterial, viral, intracellular bacterial, and noninfectious inflammatory disorders [10-15]. However, there are conflicting reports on the potential function of soluble TREM-1 (sTREM-1) in bronchoalveolar lavage (BAL) fluid as a biomarker of ventilator-associated pneumonia measured by mini-bronchoalveolar lavage or non-directed bronchial lavage (NBL) [4,16-19]. Consequently, more clinical evidence is required to establish the diagnostic role of sTREM-1 in BAL fluid. In this study we focus solely on patients with bilateral lung infiltrates, regardless of mechanical ventilation.

## Materials and methods

### Study population

**Figure 1:**
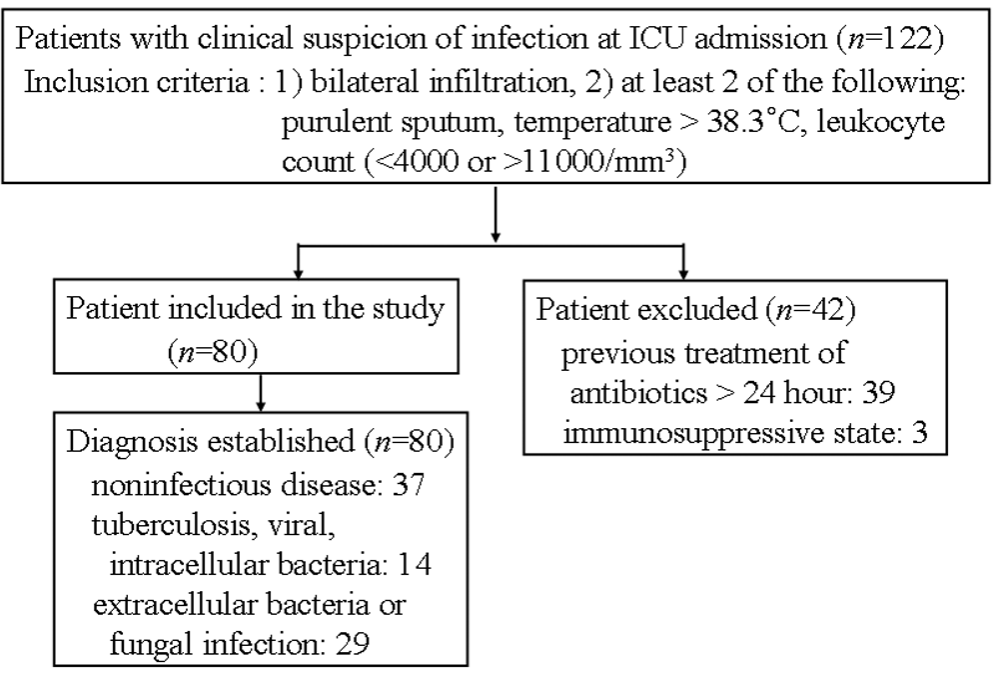
Flow diagram of patients displaying bilateral infiltration with clinical suspicion of infectious pneumonia admitted.

We enrolled 122 patients with bilateral lung infiltrates on the basis of clinical suspicion of infectious pneumonia, hospitalized in our medical intensive care unit between 1 April 2004 and 30 September 2005 (Figure 1) [2,20]. The study was approved by the Institutional Review Board of the Asan Medical Center, and written informed consent was obtained from patients or their relatives. Eligibility criteria included the following: (1) immunocompetent state, (2) age more than 18 years, (3) bilateral lung infiltrates on chest radiography at admission to the intensive care unit; and at least two of the following conditions: purulent sputum, temperature more than 38.3°C or leukocyte count of less than 4,000 or more than 11,000/mm^3 ^(4) within 24 hours of administration of the initial antibiotic therapy or immunosuppressive therapy before BAL. In total, 42 patients were excluded because of previous treatment with nonspecific broad-spectrum antibiotics (39 patients) and an immunosuppressive state (3 patients).

BAL was performed within 24 hours of admission at the intensive care unit. Additional variables recorded during admission included C-reactive protein, duration of mechanical ventilation, and length of stay in the intensive care unit. CPIS was calculated as described in a previous report [2].

Two intensivists reviewed all patient medical records and independently classified bilateral lung infiltrate diagnoses. A consensus about diagnosis was achieved in all cases. Both intensivists were unaware of the results of sTREM-1 measurements in BAL fluid. On the basis of clinical, radiological, and microbiological data, patients were assigned to one of three groups (Table 1). Group A (*n *= 37) consisted of patients with noninfectious diseases (for example acute exacerbation of interstitial lung disease, collagen vascular disease-associated lung disease, pulmonary edema, acute respiratory distress syndrome – excluding bacterial pneumonia or drug-induced lung disease). Group B (*n *= 14) included patients with tuberculosis, viral pneumonia, or atypical intracellular bacteria. Group C (*n *= 29) comprised patients with extracellular bacterial and fungal infections.

**Table 1:** Grouping of study subjects with bilateral lung infiltrates

Group	Diagnosis (*n*)
A	Acute exacerbation of interstitial lung disease (10)
(*n *= 37)	Collagen vascular disease-associated lung disease^a ^(6)
	Radiation pneumonitis (4)
	Drug-induced lung disease (3)
	Others^b^(14)
B	Atypical pneumonia (4)
(*n *= 14)	Cytomegalovirus pneumonia (3)
	Pulmonary tuberculosis (2)
	Leptospirosis (2)
	*Pneumocystis jiroveci *pneumonia (2)
	Herpes simplex virus pneumonia (1)
C	Bacterial pneumonia (27)
(*n *= 29)	Methicillin-resistant *Staphylococcus aureus *(9)
	Methicillin-susceptible *Staphylococcus aureus *(1)
	*Pseudomonas aeruginosa *(5)
	*Klebsiella pneumoniae *(2)
	*Hemophilus influenza *(1)
	ESBL *K. pneumoniae *(1)
	*Stenotrophomonas maltophilia *(1)
	Unknown (7)
	Fungal pneumonia (2)
	*Candida glabrata *(1)
	*Aspergillosis *(1)

ESBL, extended-spectrum β-lactamase.

^a^Collagen vascular disease-associated lung disease: vasculitis (*n *= 3), rheumatoid arthritis (*n *= 1), dermatomyositis (*n *= 1), and systemic lupus erythematosus (*n *= 1). ^b^Other: acute respiratory distress syndrome (*n *= 3), malignancy-associated lung disease (*n *= 3), hypersensitivity pneumonia (*n *= 2), acute eosinophilic pneumonia (*n *= 2), diffuse alveolar damage (*n *= 1), pulmonary edema (*n *= 1), sarcoidosis (*n *= 1), and postpartum hemorrhage (*n *= 1).

### Definition of disease

Patients were diagnosed with non-infectious inflammatory etiology, on the basis of clinical data, radiological signs, BAL findings, and lung biopsy. The extent of interstitial lung disease exacerbation was based on the criteria of Kondoh and colleagues [21]. These conditions included: (1) aggravation of dyspnea within 1 month, (2) hypoxemia with a ratio of arterial oxygen tension to inspired oxygen tension of less than 225, (3) newly developing pulmonary infiltrates on chest radiography, and (4) absence of apparent infection or heart disease. The diagnosis of extracellular bacterial or fungal pneumonia was based on positive blood culture or quantitative culture of BAL fluid, or a rapid response of clinical symptoms and signs to antibiotic therapy. The concentration of clinically significant microorganisms for potential diagnosis of bacterial pneumonia was more than 10^4 ^colony-forming units per ml of BAL fluid [8,22,23]. Pneumonia due to atypical intracellular bacteria (*Mycoplasma pneumoniae *and *Legionella pneumoniae*) was diagnosed on the basis of positive serologic tests showing a fourfold or greater increase in the antibody titer in paired serum samples. Diagnosis of viral pneumonia was based on clinical data, serologic tests, radiological signs [24], and biopsies.

### Assay of sTREM-1 in bronchoalveolar lavage fluid

Flexible bronchoscopy was performed on patients sedated with midazolam. BAL was performed either in the right middle lobe or the lingual segment by using 150 ml of sterile physiological saline solution in three consecutive 50 ml aliquots. The initial aspirated fluid underwent microbiological screening, and subsequent aliquots were collected for BAL analysis and sTREM-1. BAL fluid was subsequently filtered through sterile gauze to remove mucus, and then centrifuged at 500 *g *and 4°C for 15 min to obtain the cell pellet. The supernatant was centrifuged, separated, and stored as aliquots at -80°C until further analysis.

The sTREM-1 concentration in BAL fluid samples was measured with a DuoSet enzyme-linked immunosorbent assay kit (R&D Systems, Minneapolis, MN, USA) [16,18] consisting of a capture antibody (mouse anti-human TREM-1), standard antibody (recombinant human TREM-1), and detection antibody (biotinylated goat anti-human TREM-1). Intra-assay and inter-assay coefficients of variation were 2.8% and 5.2%, respectively.

### Statistical methods

Categorical data were compared by using Fisher's exact test, and continuous data were compared with the Kruskal–Wallis test. To evaluate the diagnostic value of data we used a logistic regression model. Receiver operating characteristic (ROC) curves were constructed to illustrate the various cutoff values of sTREM-1, CPIS, and neutrophil count in BAL fluid. Continuous variables are expressed as mean ± SEM, and two-tailed *P *values of less than 0.05 were considered statistically significant. All data were analyzed with SPSS version 11.0 (SPSS Inc, Chicago, IL, USA).

## Results

### Patient characteristics

Characteristics of the study subjects are shown in Table 2. Groups A and B displayed similar clinical and laboratory features. In contrast, group C displayed neutrophilia in BAL fluid and a high CPIS score compared with group A (Table 3). Pathogens were cultured in 76% of samples from group C patients (Table 1).

**Table 2:** Characteristics of patients with bilateral lung infiltrates

Characteristic	Group A (*n *= 37)	Group B (*n *= 14)	Group C (*n *= 29)
Age, years	57.8 ± 2.8	63.7 ± 3.4	61.7 ± 3.3
Sex, M:F	22:15	10:4	23:6
APACHE II score at entry	18.1 ± 1.0	16.8 ± 1.4	21.6 ± 1.4^a^
Co-morbidities, *n*			
Malignancy	12		6
Chronic heart disease	2	1	1
Chronic lung disease		2	3
Chronic liver disease		1	2
Chronic renal disease	1		1
Endocrinologic disease	4		
Neurologic disease	2		6
Transplantation		1	1
Duration of mechanical ventilation, days	11.3 ± 1.7	10.2 ± 3.6	11.2 ± 2.7
Length of stay in ICU, days	16.3 ± 2.2	10.9 ± 3.1	18.3 ± 2.7^a^
Mortality in ICU, percentage	40.5	28.6	42.9

Results are presented as mean ± SEM. Group A: noninfectious; group BI virus, tuberculosis, intracellular bacteria; group C: extracellular bacteria, fungi. APACHE, Acute Physiology and Chronic Health Evaluation; ICU, intensive care unit.

^a^*P *< 0.05 versus group B.

**Table 3:** Characteristics of the three groups of patients with bilateral lung infiltrates at enrollment

Characteristic	Group A (*n *= 37)	Group B (*n *= 14)	Group C (*n *= 29)
CPIS	6.4 ± 0.4	7.4 ± 0.4	8.8 ± 0.4^a^
C-reactive protein, mg/dl	11.0 ± 1.5	14.0 ± 2.0	12.6 ± 2.6
BAL fluid findings, percentage			
Neutrophils	34.6 ± 8.6	36.0 ± 15.0	66.7 ± 7.0^a^
Alveolar macrophages	29.3 ± 8.0	28.7 ± 6.4	15.7 ± 4.1^a^
Lymphocytes	22.5 ± 6.1	24.5 ± 10.9	11.2 ± 5.3
Eosinophils	7.0 ± 3.4	8.2 ± 5.2	3.7 ± 2.6
sTREM-1, pg/ml	92.8 ± 10.7	92.9 ± 20.0	521.2 ± 94.7^a ^^b^

Results are presented as mean ± SEM. Group A: noninfectious; group B: virus, tuberculosis, intracellular bacteria; group C: extracellular bacteria, fungi. CPIS, Clinical Pulmonary Infection Score; BAL, bronchoalveolar lavage; sTREM-1, soluble triggering receptor expressed on myeloid cells-1.

^a^*P *< 0.05 versus group A; ^b^*P *< 0.05 versus group B.

**Figure 2:**
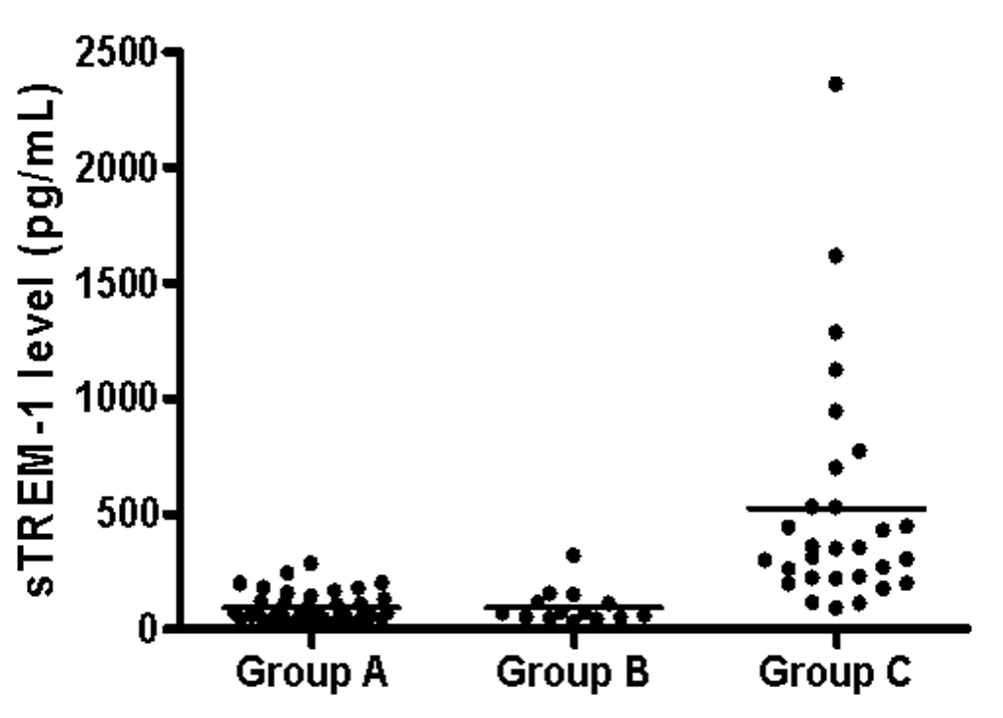
Concentration of sTREM-1 in bronchoalveolar lavage fluid of patients with bilateral lung infiltrates. Group A, noninfectious inflammatory disease; group B, atypical pneumonia, viral pneumonia, and tuberculosis; group C, bacterial or fungal pneumonia. Individual values are plotted; bars represent the median values. sTREM-1, soluble triggering receptor expressed on myeloid cells-1.

The sTREM-1 concentration was significantly elevated in group C (521.2 ± 94.7 pg/ml), compared with groups A (92.8 ± 10.7 pg/ml, *P *< 0.05) and B (92.9 ± 20.0 pg/ml, *P *< 0.05) (Figure 2). Subgroup analysis of group C (community-acquired pneumonia, nosocomial pneumonia, and ventilator-associated pneumonia) disclosed that sTREM-1 concentrations were not significantly different between the three subgroups (Additional File 1).

### Diagnostic value of the sTREM-1 assay

**Figure 3:**
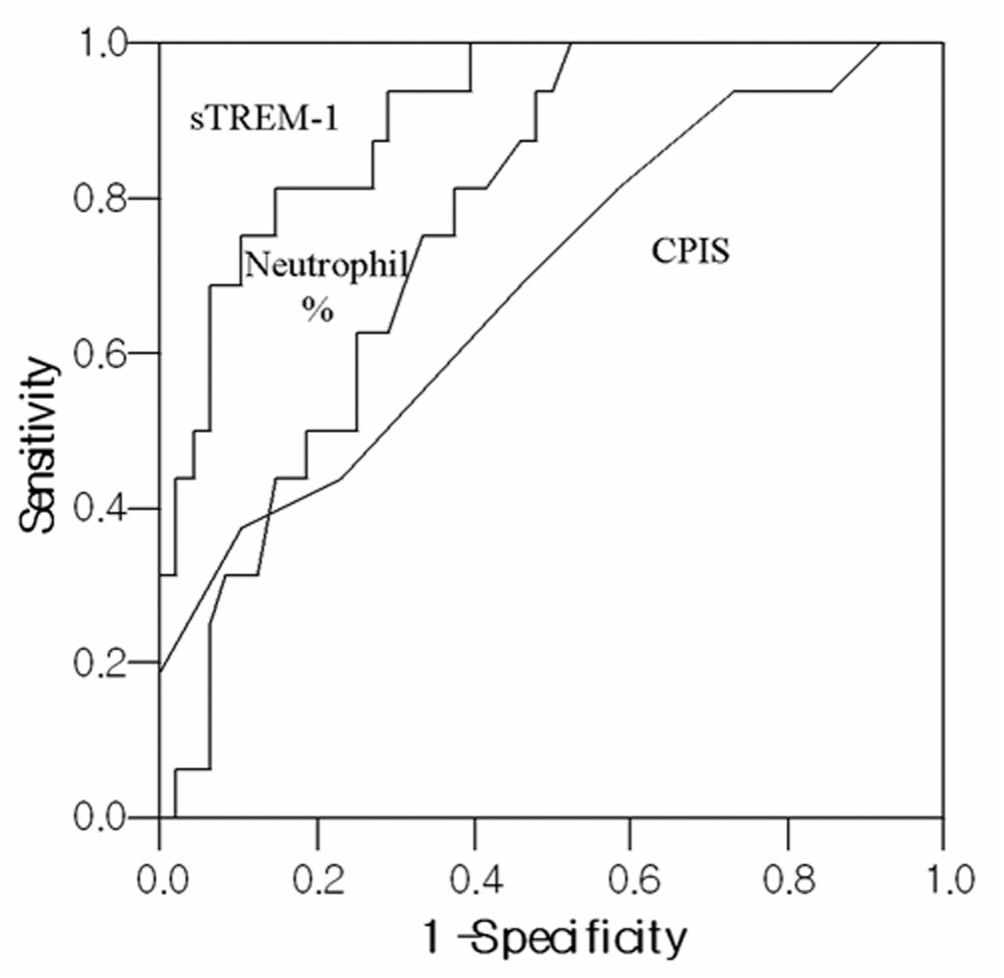
ROC curve of sTREM-1, neutrophil percentage in BAL fluid, and CPIS for diagnosis of bacterial and fungal pneumonia. Areas under the receiver operating characteristic (ROC) curve were 0.91 (95% confidence interval (CI), 0.83 to 0.98; *P *= 0.000) for soluble triggering receptor expressed on myeloid cells-1 (sTREM-1), 0.77 (95% CI 0.54 to 0.84; *P *= 0.001) for percentage of neutrophils in bronchoalveolar lavage fluid, and 0.69 (95% CI 0.54 to 0.84; *P *= 0.023) for Clinical Pulmonary Infection Score (CPIS).

We employed ROC curve analysis (Figure 3) to determine whether the sTREM-1 concentration in BAL fluid can be used to discriminate between the possible causes of bilateral lung infiltrates. The area under the ROC curve, using sTREM-1 to differentiate between the presence and the absence of bacterial and fungal pneumonia, was 0.91 (95% confidence interval (CI) 0.83 to 0.98; *P *< 0.001). A sTREM-1 cutoff value of 184 pg/ml correlated with sensitivity and specificity values of 86% (95% CI 72.9 to 99.6) and 90% (95% CI 81.8 to 98.7), respectively. A positive likelihood ratio of 8.79, a negative likelihood ratio of 0.11, and an odds ratio of 57.50 (95% CI 14.15 to 233.66) were calculated. At a level of 184 pg/ml or higher, sTREM-1 was detected in BAL fluid from 25 of 29 patients with bacterial or fungal pneumonia (sensitivity 86%; 4 false-negative results), 4 of 37 patients with noninfectious inflammatory disease (4 false-positive results), and 1 of 14 patients with atypical pneumonia, viral pneumonia, or tuberculosis (1 false-positive result). Three of the five false-positive cases showed diffuse alveolar hemorrhage in BAL fluid without reference to infection. On exclusion of patients with diffuse alveolar hemorrhage, the sTREM-1 cutoff value of 184 pg/ml yielded sensitivity and specificity values of 92% (95% CI 80.6 to 100) and 95% (95% CI 87.6 to 100), respectively.

A multiple logistic regression analysis showed that the sTREM-1 level (184 pg/ml) in BAL fluid is an independent predictor of bacterial or fungal pneumonia with an odds ratio of 59.742 (95% CI 6.610 to 539.930) (Table 4). No correlation was evident between the neutrophil count and sTREM-1 in BAL fluid (*r *= 0.214, *P *= 0.069).

**Table 4:** Multiple logistic-regression analysis of factors used for differential diagnosis of bacterial or fungal pneumonia

Predictor	Odds ratio	95% CI	*P*
BAL fluid sTREM-1 level ≥ 184 pg/ml	59.742	6.610–539.930	0.000
BAL neutrophils ≥ 60%	11.517	1.227–108.084	0.032
CPIS > 6	0.484	0.068–3.459	0.470

BAL, bronchoalveolar lavage; sTREM-1, soluble triggering receptor expressed on myeloid cells-1; CPIS, Clinical Pulmonary Infection Score; CI, confidence interval.

## Discussion

The main findings of this study are that sTREM-1 concentration can be used effectively in the diagnosis of bacterial or fungal pneumonia in patients with bilateral infiltration, and that a modified CPIS of more than 6 is not a valid diagnostic indicator of pneumonia using multivariate analysis.

In cases where patients displayed localized consolidation on a chest radiogram, diagnosing pneumonia is less difficult than identifying the cause of bilateral infiltration. The appropriate diagnosis of bilateral lung infiltrates in critically ill patients is crucial but difficult. In many cases, bilateral lung infiltrates are associated with noninfectious inflammatory diseases. Although previous reports show that a CPIS of more than 6 indicates a high likelihood of pneumonia, its diagnostic accuracy in bilateral lung infiltrates is controversial [2,4]. A CPIS value greater than 6 was also a useful screening tool (82% sensitivity) in the present study, but its specificity for differential diagnosis of bilateral bacterial pneumonia was low (39%). In contrast to our findings, Gibot and colleagues reported that CPIS could be effectively applied to differentiate between patients with and without pneumonia (including community-acquired pneumonia). Our study included 68.4% of patients with a CPIS of more than 6, compared with 49% of patients in Gigot's study. It therefore seems that a CPIS of more than 6 is not an efficient factor in the diagnosis of pneumonia with bilateral lung infiltrates.

Several earlier studies have focused on sTREM-1 in patients with pneumonia. The present study, however, involved only bilateral lung infiltration and took into consideration several cases of acute exacerbation of interstitial lung disease, which is difficult to distinguish from superimposed pneumonia. Although some patients with bilateral infiltration were analyzed by Gibot and colleagues, this condition was not the focus of the earlier study. In addition, Gibot and colleagues did not include patients with acute exacerbation of idiopathic pulmonary fibrosis or viral pneumonia [4]. In another study, Richeldi and coworkers did not include patients with pneumonia caused by 'atypical' intracellular pathogens or fungi or those admitted to the intensive care unit, and employed cytofluorimetric analysis [25]. Our results not only confirm several previous findings but also provide additional information.

Here we show that the sTREM-1 level in BAL fluid constitutes an independent factor in the differential diagnosis of bacterial or fungal pneumonia at a cutoff level higher than 184 pg/ml. Determann and coworkers reported that at a cutoff value of 200 pg/ml, sTREM-1 levels in NBL fluid in ventilator-associated pneumonia yielded diagnostic sensitivity and specificity values of 75% and 84%, respectively [16]. This study was performed with bronchoscopic BAL fluid instead of NBL fluid. Previous data were obtained primarily with NBL fluid, which may differ from BAL fluid in terms of specific characteristics. However, the sTREM-1 levels were not significantly different between BAL fluid and NBL fluid.

In the present study we observed no correlation between neutrophil counts and sTREM-1 levels in BAL fluid, indicating that activation of neutrophils and amplification of the inflammatory response occur via different mechanisms. sTREM-1 may have a role in acute inflammation characterized by an exudate of neutrophils and monocytes. Moreover, lipopolysaccharides, bacteria, and fungi upregulate sTREM-1 expression [10,12-15].

The present study has several limitations. First, because most of the false-positive results in sTREM-1 levels involved diffuse alveolar hemorrhage, which was not included in other investigations [4,16,18,25], the utility of sTREM-1 in this group remains to be determined. Second, some patients may have suffered from noninfectious inflammatory disease combined with infection, although two blinded investigators determined each patient's diagnosis without knowledge of the sTREM-1 concentration. Third, the sTREM level measured in BAL fluid is lower as a result of dilution and may differ from the actual concentrations in some patients, although we performed exactly the same technique and retrieved similar volumes in the three groups (data not shown). Finally, cases of fungal pneumonia were rare.

## Conclusion

The sTREM-1 level in BAL fluid from patients with bilateral lung infiltrates is a potential marker for the differential diagnosis of pneumonia due to extracellular bacteria. We propose that the sTREM-1 level (184 pg/ml or more, versus less than 184 pg/ml) is a more useful marker than clinical criteria in refining the diagnostic spectrum (bacterial infection versus others) in patients presenting bilateral lung infiltrates.

## Key messages

• The sTREM-1 concentration in BAL fluid is an independent predictor of bacterial or fungal pneumonia in patients with bilateral lung infiltrates, and a cutoff value of more than 184 pg/ml yields a diagnostic sensitivity of 86% and a specificity of 90%.

• A modified Clinical Pulmonary Infection Score of more than 6 does not show clinical usefulness for the diagnosis of pneumonia in patients with bilateral lung infiltrates.

• The sTREM-1 level may be applied as a useful marker for the differential diagnosis of bilateral lung infiltrates.

## Abbreviations

BAL = bronchoalveolar lavage; CI = confidence interval; CPIS = Clinical Pulmonary Infectious Score; NBL = non-directed bronchial lavage; ROC = receiver operating characteristic; sTREM-1 = soluble triggering receptor expressed on myeloid cells-1; TREMs = triggering receptors expressed on myeloid cells.

## Competing interests

The authors declare that they have no competing interests.

## Authors' contributions

HJW and HSB initiated the study. KYS, LCM, OYM, STS, LSD, KWS, KDS, and KWD participated in patient management. HJW and HSB analyzed the data. All the authors contributed to and approved the final manuscript.

## Supplementary Material

Additional file 1 file containing two supplementary tables. Click here for file
