# Supplementary material for: Diagnostic utility of the soluble triggering receptor expressed on myeloid cells-1 in bronchoalveolar lavage fluid from patients with bilateral lung infiltrates
Source: Crit Care. 2008 Jan 19;12(1):R6. doi: 10.1186/cc6770 (PMC2374623; doi:10.1186/cc6770)
Supplement: Additional file 1 — file containing two supplementary tables. [file cc6770-S1.doc]

Supplement 1. Subgroup analysis of Group C

|  | Community- acquired pneumonia  (*n* = 8) | Nosocomial pneumonia  (*n* = 16) | Ventilator- associated pneumonia  (*n* = 5) |
| --- | --- | --- | --- |
| Clinical pulmonary infection score | 9.6 ± 0.8 | 8.3 ± 0.6 | 9.4 ± 0.9 |
| C-reactive protein, mg/dL | 12.5 ± 2.8 | 14.3 ± 4.5 | 7.3 ± 1.6 |
| sTREM-1, pg/mL | 493.2 ± 158.3 | 578.1 ± 150.7 | 383.5 ± 116.3 |

Supplement 2. Univariate analysis of factors used for the differential diagnosis of bacterial or fungal pneumonia

| Predictor | Odds ratio | 95% CI | *P* value |
| --- | --- | --- | --- |
| BAL sTREM-1 level≥ 184 pg/mL | 57.500 | 14.150–233.659 | 0.000 |
| BAL neutrophils ≥ 60% | 5.120 | 1.526–17.173 | 0.008 |
| Clinical pulmonary infection score>6 | 2.968 | 0.970–9.083 | 0.057 |
| C-Reactive Protein | 1.006 | 0.965-1.049 | 0.776 |
